# "Diet and Hygiene" Campaign

**Published:** 1921-03-12

**Authors:** 


### "Diet and Hygiene" Campaign.

### AN APPEAL TO THE GOVERNMENT.

A memorial has been sent to the Minister of Health by medical, scientific, health, labour, and philanthropic associations, representing thousands of men and women in all classes of society in various parts of the country. These organisations consider that the "preventable suffering and avoidable disablement" described by the Chief Medical Officer of the Ministry would be diminished by a general knowledge of diet and hygiene, and that it would also help to prevent the national disgrace of not less than a million children of school age being "so physically or mentally defective or diseased as to be unable to derive reasonable benefit from the ordinary form of education which the State provides." The memorial asks what has been done, and what will be done in spreading information about diet and hygiene, as it was officially stated in Grand Committee that this would be "one of the first duties" of the Ministry of Health. The Societies, without advocating any special system of diet, suggest that much good would be accomplished by directing attention to the cheap supplies of vitamine and other essential food substitutes, which can be secured by utilising whole cereals and pulse, fruits, salads, and vegetables. Since the above appeal was communicated to the Ministry, Dr. Addison, in reply to a question in the House of Commons, said he was considering the practicability of issuing a pamphlet on the lines here indicated, but he suggested difficulties which we think might very easily be avoided. We are glad to see that pressure is to be continued in the House, and that, almost at the moment of writing, another question has been put down on the same subject.

All persons interested in the public health should support to the best of their ability the movement for a sensible reform in the national diet. There is still a certain amount of prejudice against the kind of propaganda work carried on by a body like the Bread and Food Reform League, because for a long time food reformers have been associated in the public mind with "cranks" and faddists. Even now a man who calls himself a vegetarian is regarded with almost as much objective curiosity as a Christian Dawnist, or a faith-healer, or a "Pussyfoot" zealot. Yet, while there are "food cranks" of a type that may be numbered among the phenomena of the age, it would be a great mistake to confuse their idiosyncrasies with a genuine and rational movement which is seeking to banish the baffling and appalling ignorance among the masses about food values, and to create an intelligent interest in the choice and preparation of domestic meals. It is not too much to say that half the minor ailments of life, much of the ill-temper of the middle-aged, and a good deal of serious ill-health are produced solely by the artificial and badly cooked food that most people are content to eat.

Recent investigation has shown how, by a careful attention to the elementary rules of hygiene and a proper distribution of food values whose effect can now be carefully calculated, a great step forward could be taken in the national health. Voluntary organisations, by the issue of pamphlets and by lecture demonstrations, have done something to interest the general public in its own behalf. But "such a being as man in such a world as this," while he will remain comparatively unimpressed by the enthusiastic appeals of well-meaning individuals, will give more serious attention to a presentation of the case that comes with official sanction and authority. Therein lies the importance of inducing the Government to spread the desired information in simple form, avoiding at the same time controversial assertions that may excite the anger of controversially minded experts. We remember a useful pamphlet sent out in 1915 by the Board of Education, which contained a number of suggestions for simple and nourishing meals for the home. Its title was "Economy in Food," and it was admirable in its straightforward presentation of the facts. We do not know whether this pamphlet, known at the time as Circular 917, is still available to the public; if not, it might easily be made the basis of a further issue, containing the accepted results of up-to-date investigations. By the careful choice of nourishing food, and by avoiding all waste, the modern housewife can at once improve the health of her household, and make a real saving in the domestic budget. The child-life of the country is bound up in the necessity of preventing the deterioration and adulteration of meal, flour, and bread, and other necessaries of existence. We hope later to return to this subject in more detail; but in the meantime it is satisfactory to know that the food reformers inside and outside Parliament do not intend to let the matter drop until they have extracted from the Government a definite promise to tackle the problem in a practical manner.

